# Muscle Activation Timing and Synergies During End-Effector Robot-Assisted Gait: A Surface EMG Study

**DOI:** 10.3390/s26216960

**Published:** 2026-11-04

**Authors:** Matteo Cioni, Maria Stella Valle, Laszlo Kovacs, Daniela Vorazzo, Caterina Sergi, Gianfranco Bosco

**Affiliations:** 1 Laboratory of Neuro-Biomechanics, Section of Physiology, Department Biomedical and Biotechnological Sciences, University of Catania, 95123 Catania, Italy; m.valle@unict.it; 2 Center of Rehabilitation, A.A. Villa Sandra, S. Giovanni La Punta, 95037 Catania, Italy; kovacslaszloft@gmail.com (L.K.); caterinasergi@tiscali.it (C.S.); 3 BTS Bioengineering, 20024 Garbagnate Milanese, Italy; daniela.vorazzo@btsbioengeenering.com; 4 Department of Systems Medicine, Center for Space Biomedicine, University of Rome Tor Vergata, 00133 Roma, Italy; bosco@med.uniroma2.it

**Keywords:** gait, robotic gait cycle, end-effector robot, surface electromyography, Non-negative Matrix Factorization, cluster analysis, dynamic electromyography

## Abstract

**Highlights:**

**What are the main findings?**

- We have shown the topography of lower limb muscle activation timing during walking with an end-effector robot.
- Three distinct temporal muscle synergies emerge during the robotic gait cycle.

**What are the implications of the main findings?**

- We propose a protocol-specific reference framework for the timing of lower-limb muscle activation against which pathological patterns could be compared.
- The temporal muscle activation patterns of the lower extremities appeared to be preserved between passive and active-assisted robotic locomotion.

**Abstract:**

Surface electromyographic (SEMG) patterns during robot-assisted walking are still poorly documented. This study aimed to characterize the timing and synergies of muscle activations of the lower extremities during locomotion assisted with an end-effector robot. Healthy subjects walked at 1.8 km/h under two modes: passive and active with a full body weight load. The robotic gait cycle (RGC) was identified by detecting the flexion-extension phases of the right lower limb. SEMG signals were recorded from eight lower limb muscles, time-normalized with respect to the RGCs and averaged across 10 RGCs. Non-negative Matrix Factorization (NMF) identified three basic waveforms (factors) explaining 90.6% of the variance in the dataset of SEMG waveforms corresponding to three distinct muscle synergies: rectus femoris–vastus lateralis–vastus medialis (extension phase), gastrocnemius medialis–soleus–peroneus longus (mid-flexion phase), and tibialis anterior–biceps femoris longus (transition between extension and early flexion phases). Cluster analysis on the cosine distance matrix of the NMF factor coefficients further validated the three distinct muscle groupings. Temporal activation patterns were seemingly preserved between passive and active conditions, suggesting similar underlying neuromuscular organization. This study supports the use of clinical gait analysis, particularly with SEMG, to assess neuromuscular patterns of neurological patients before, during, and after robotic rehabilitation.

## 1. Introduction

Assistive devices, such as treadmills, have been employed extensively by experimental studies to investigate the neural control of locomotion. These experimental paradigms allow the activation of central pattern generators (CPGs) located in the spinal cord through the stimulation of peripheral sensory receptors in muscles and joints of hindlimbs [1]. Additional investigations have explored the contribution of subcortical locomotor regions to the walking of decerebrates while suspended on a treadmill, demonstrating their role in modulating locomotor cadence and rhythmicity [2]. More recently, Valle et al. [3] provided experimental evidence that during robotic locomotion, the fundamental kinematic framework observed under purely passive conditions is preserved during active stepping.

Along the same line, treadmill-based locomotion paradigms have been widely used to investigate the organization of CPGs and the underlying electromyographic patterns in healthy individuals [4], the plastic adaptations that occur in patients with spinal cord injury [5], and the effects of pharmacological interventions on locomotor recovery [6]. Collectively, these studies have provided the neurophysiological foundation for the development of rehabilitation interventions based on robotic assistive technologies.

During the last two decades, robotic devices have been increasingly adopted in neurorehabilitation. Both exoskeleton-based systems and end-effector robotic devices have been evaluated in several clinical studies [7,8]. These investigations consistently demonstrated the effects of robotic-assisted gait training (RAGT) in the rehabilitation of individuals with spinal cord injury [9,10,11,12], post-stroke gait impairments [13,14], post-stroke in the elderly [15], and cerebral palsy [16], particularly when RAGT is integrated into structured physiotherapy programs. In most of these clinical studies, the effectiveness of RAGT has been evaluated primarily through ordinal clinical scales, such as the Fugl-Meyer Assessment, the Stroke Impact Scale, and the Impairment Scale Clinical Examination, or through functional performance tests such as the 10-m walking test. Although these outcome measures provide important clinical information, they offer limited insight into the underlying neuromuscular mechanisms involved in assisted robotic locomotion (ARL).

Despite the growing adoption of robotic devices in neurorehabilitation, the neuromuscular mechanisms underlying ARL are not yet fully understood. To date, only a few studies have addressed this issue in both healthy individuals and neurological populations. Hidler et al. [17] reported that walking with an exoskeleton resulted in significant differences in spatial and temporal muscle activation patterns compared to walking on a treadmill. Similarly, Coenen et al. [18] reported a reduction in surface electromyographic (SEMG) amplitude during robotic walking in both post-stroke patients and healthy controls. More recently, Bellitto et al. [19] compared SEMG activity during end-effector-assisted locomotion and overground gait, showing that muscle activation patterns during ARL appear to be more influenced by the mechanical characteristics of the device than the assistance provided.

Identifying SEMG activation patterns under ARL is necessary to interpret neuromuscular adaptations during rehabilitation and to identify pathological deviations in clinical populations. Therefore, a deeper understanding of the neuromuscular organization of robotic locomotion in healthy individuals is appropriate to establish physiological benchmarks against which pathological patterns observed during rehabilitation can be interpreted.

In this respect, data reduction analysis methods such as Principal Components Analysis (PCA) or Non-negative Matrix Factorization (NMF) have been widely used to investigate muscle synergies quantitatively during walking in both physiological and pathological conditions [4,20,21], and therefore could represent useful tools to characterize potential muscle synergies occurring during ARL. The present study aimed to characterize in a cohort of subjects the temporal organization of SEMG activation patterns during ARL with an “end effector robot”. Secondly, we hypothesized that lower limb muscles are synergistically active, in the time domain, according to the different phases of the robotic gait cycle (RGC). Third, we wanted to verify whether the timing of muscle activations was influenced differently by the passive or active modes of ARL.

## 2. Materials and Methods

### 2.1. Participants

Eleven healthy women (age range: 23–50 yrs, mean age: 28.6 ± 8.7 yrs, mean height: 1.64 ± 0.05 m; mean weight: 55.09 ± 4.50 kg; mean BMI: 19.99 ± 1.35) were consecutively recruited. All participants provided written informed consent. They were not involved in competitive sports activities. They underwent a clinical examination to assess the range of motion of the lower limbs, muscle strength, and leg length discrepancy. The right lower limb was dominant for all participants. No participant had known orthopedic, neurological, or gait disorders. They were dressed in shorts and gym shoes. Given the exploratory nature of the study, a sample size of 10 subjects was identified based on previous studies with a similar methodology (ARL with SEMG), which ranged from 3 to 10 subjects [17,22,23,24].

The study protocol was approved by the Catania 2 Ethics Board (Presidio Ospedaliero Garibaldi Nesima-Catania, Catania, Italy) n. prot. 250/CEL (date of approval: 24 October 2025). All procedures were carried out according to the Declaration of Helsinki (1975 and revised 2013).

### 2.2. Intervention

Recordings of ARL were performed at the Rehabilitation Center A.A. Villa Sandra (S. Giovanni La Punta, Catania, Italy), using an end-effector robotic device (LEXO, GmbH, Tyromotion, 8020 Graz, Austria).

Sessions were performed with full body weight load (100%) measured while subjects were standing on the robotic device, supporting their own weight without lift assistance.

The recordings were performed under two experimental conditions: (A) passive mode; (B) active mode. For the passive mode, subjects were instructed to follow the movement imposed by the robot, while for the active mode, they were instructed to push alternately (right–left) over the anterior footplate by applying a load of 50% of body weight.

None of the subjects had previous experience with ARL. At their arrival in the laboratory, they were instructed about the exercise to be performed by first showing another subject walking with the robotic device and then allowing them to familiarize themselves with the device and the type of locomotion. An experienced and certified physical therapist (one of the authors, L.K.) set up the device and assisted each subject.

Based on a preliminary evaluation aimed at identifying the walking parameters that provided a comfortable robotic gait, a step length of 60 cm and a maximum walking speed of 1.8 km/h were selected for all participants. Following a 10-min familiarization period, participants performed locomotor training in two sessions (passive and active modes), presented in a randomized order, each lasting 5 min. SEMG recordings were collected for 1 min during the intermediate phase of each session. The compliance of the subjects was monitored by observing the fluency of the robotic locomotion. In fact, if a subject could not follow robotic movements (passive mode) or push the anterior footplate (active mode) with a pre-fixed load of 50%, locomotion became evidently non-fluent. If performance was not considered adequate, the trials were rejected based on two a priori criteria: firstly, clinical observation, and secondly, accurate offline check of each slope of the robotic gait cycle. In general, trials were rejected only sporadically, just at the beginning of the trial itself, and soon after the trial was restarted.

### 2.3. Definition of the Robotic Gait Cycle

**Figure 1:**
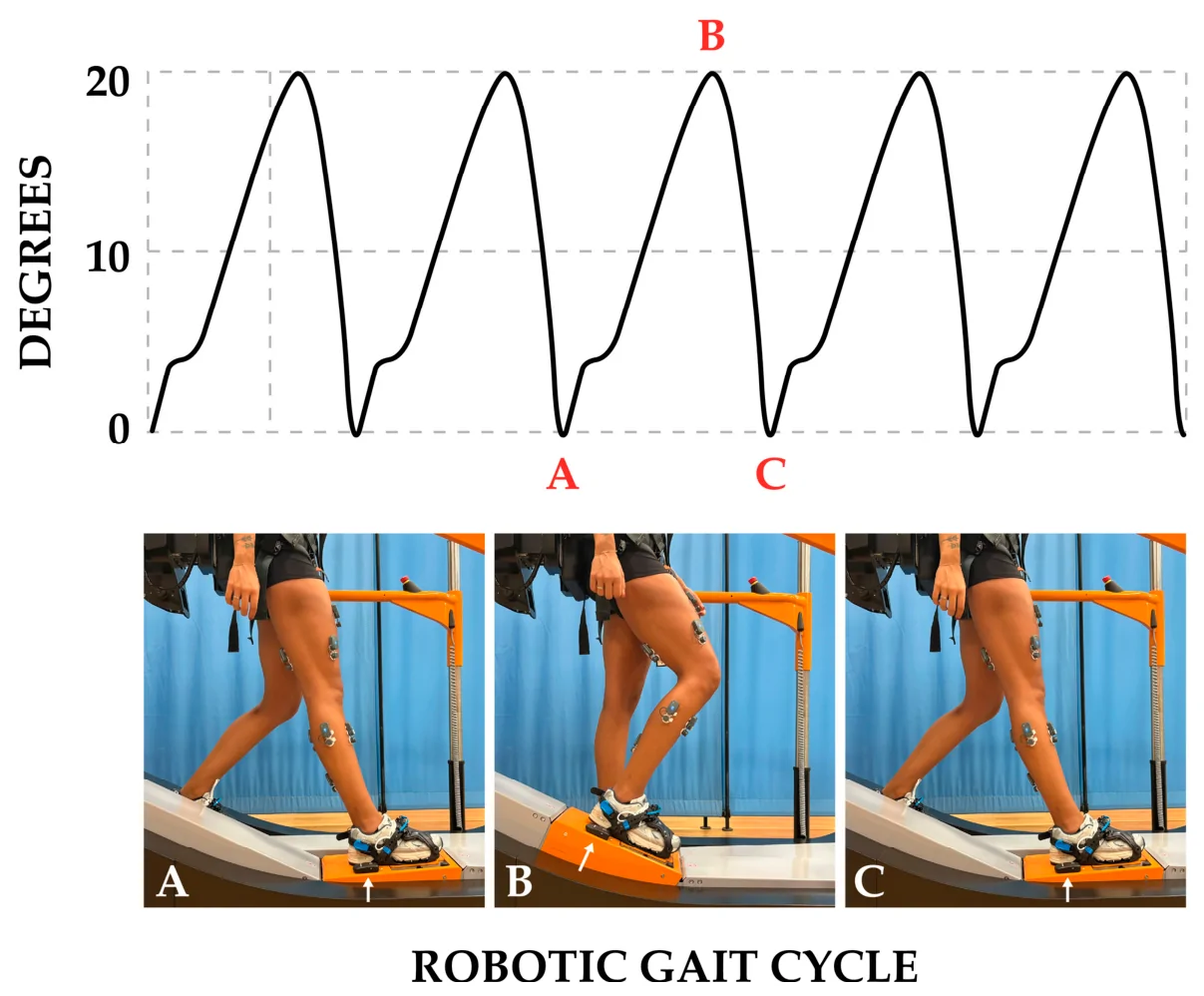
(**Top**): kinematics profiles of right footplate motions recorded by a wearable sensor applied over the right footplate. (**A**–**C**): robotic gait cycle; (**A**–**B**): flexion phase; (**B**–**C**): extension phase. (**Bottom**): position of the right knee at the onset of RGC (**A**), at the offset of RGC (**C**), and at the transition phase from the flexion phase to the extension phase (**B**). Arrow: position of the wearable sensor.

A wearable sensor (G-Sensor, BTS Bioengineering, 20024 Garbagnate Milanese, Milano, Italy), which incorporates an accelerometer and a gyroscope, was positioned on the right footplate near the external face of the heel of the right lower limb. The sensor allowed measurement of the position (degrees) of the footplate over which the right foot was strapped. The calibration of the wearable sensor was performed while the subject was standing on the locomotor device with the footplates in a horizontal position. The onset and offset of the RGC corresponded respectively to the position of the right footplate, slightly posteriorly inclined (−1°), and the angle of the ankle and foot, slightly opened in plantar flexion of a few degrees (Figure 1).

Based on the motion of the knee joint, two distinct phases of the RGC were defined: a long flexion phase, during which the robotic device induced a knee flexion, and a short extension phase, during which the knee was extended to its initial position. The two phases, along with their corresponding kinematic profiles of right footplate motion, are shown in Figure 1.

Sensor signals were synchronized with electromyographic recordings, and both were wirelessly transmitted to a personal computer for offline analysis, using EMG Analyzer software Version 2.10.44.0 (BTS Bioengineering, Italy). The software was also used to compute the temporal parameters of the RGC, including the cycle duration (s), flexion and extension phase percentages, phase duration (s), and cadence (steps/min).

### 2.4. Acquisition and Processing of SEMG

SEMG activity was recorded exclusively from the right lower limb. This choice was based on two considerations: (1) all participants were healthy and did not show anatomical or clinical differences between the lower limbs; and (2) previous studies have consistently demonstrated that, in healthy individuals, the right and left lower limbs exhibit similar SEMG activation patterns during walking [25]. SEMG signals were recorded from the m.m. tibialis anterior (TA), gastrocnemius medialis (GM), soleus (SOL), peroneus longus (PL), rectus femoris (RF), vastus lateralis (VL), vastus medialis (VM), and biceps femoris caput longus (BF).

Disposable electrodes (ARBO, Kendall, Cardinal Health, Dublin D18 X5K7, Ireland) were placed according to the SENIAM guidelines [26] and Blanc et al. [27] and wirelessly connected to the FREE EMG 1000 system (BTS Bioengineering, 20024 Garbagnate Milanese, Milano, Italy). Signals were sampled at 1000 Hz, band-pass filtered (4th order Butterworth, 20–400 Hz, bidirectional), and amplified. For each participant, the rectified and low-pass filtered (4th order Butterworth, cut-off frequency 5 Hz, bidirectional) SEMG signals of 10 RGCs per condition were extracted and time-normalized to 500 points. The mean and standard deviation values were calculated across the 10 RGCs.

### 2.5. Data Analysis

To determine the onset and offset of muscle activity bursts during the RGCs, we applied a double-threshold method to the rectified, low-pass-filtered, and averaged SEMG traces across consecutive RGCs. The double threshold method, according to Goffredo et al. [28] and Paoloni et al. [29], consisted of an amplitude threshold of 20% of the minimum–maximum amplitude excursion combined with a temporal threshold of 50 ms during which the amplitude of the SEMG signal had to remain above the 20% threshold. An expert clinical gait analyst performed post hoc quality checks on the SEMG signals, blindly with respect to experimental conditions and muscles, to identify potential artifacts and validate the outcomes of the two-step SEMG threshold algorithm (one of the authors, M.C.).

An electromyographic activation pattern that was consistently observed in all participants within a given segment of the RGC was considered representative, and its median value was calculated. A Wilcoxon signed-rank test was conducted to compare the two conditions (passive vs. active) for the duration of activations during RGC. The statistical significance cut-off was set to *p* < 0.05, corrected for multiple comparisons by using the Benjamini–Hochberg false discovery rate (FDR) method.

To analyze more quantitatively the mean SEMG temporal profiles across muscles, stepping conditions, and participants, we applied a Non-negative Matrix Factorization (NMF) [30] to the entire dataset of mean SEMG recordings, that is, a 500 × 176 matrix comprising 176 SEMG waveforms (8 muscles × 11 subjects × 2 conditions) with 500 time-samples. Since the study focused on the temporal features of SEMG activity, prior to submitting the SEMG waveforms dataset to the NMF analysis, we normalized them to their respective maxima, thereby preventing the factorization from being dominated by the SEMG signals with the largest amplitude. Factorization was performed with the R package “NMF”, according to the multiplicative update algorithm proposed by Lee and Seung [30] and the R default parameters for this method, namely, Frobenius norm metric, random seeding, stationarity of the connectivity matrix as stopping criterion, and number of runs = 100 and maximal number of iterations = 1000. To determine the optimal number of basis waveforms (factors), NMF models with one to eight factors were calculated, and the percentage of variance accounted for (VAF) was evaluated for each solution. The final number of factors was selected with the elbow criterion, that is, by identifying the point beyond which additional factors resulted in only marginal increases in VAF. Following extraction of NMF factors, the similarity between the vectors of factor coefficients was quantified by calculating pairwise cosine distances using the package “lsa” in R. The resulting cosine distance matrix was then submitted to a Partitioning Around Medoids (PAM) cluster analysis using the R “cluster” package and considering the cosine distance matrix as a dissimilarity matrix. The optimal number of clusters was identified with the silhouette method implemented by the “fviz_nbclust” function of the “factoextra” R package, by testing for cluster solutions comprised between *n* = 2 and *n* = 8 and by verifying cluster stability with 1000 Montecarlo bootstrap samples for each cluster solution (nboot option = 1000). In brief, the silhouette width, which quantifies the degree of similarity of an observation to its assigned cluster relative to other clusters, was calculated for the different clustering solutions. Then, the number of clusters that yielded the highest average silhouette width was considered optimal. This cluster analysis allowed the identification of muscle groups with similar weighting patterns of NMF basis waveforms, denoting potential temporal muscle synergies. Finally, we compared the SEMG temporal profiles between passive and active stepping conditions in the space defined by the factors extracted with the NMF, by applying, for each NMF factor, Wilcoxon paired sign-rank tests to the distributions of the NMF factor coefficients of each muscle in the passive and active conditions. The statistical significance cut-off was set to *p* < 0.05 corrected for multiple comparisons across the eight muscles by using the Benjamini–Hochberg false discovery rate (FDR) method. All statistical analyses were performed using the R statistics software (ver. 4.5.3) running with the RStudio graphical interface (Posit software, ver. 2626.0.1.0+392).

## 3. Results

### 3.1. Robotic Gait Cycle

The flexion phase spanned from 0 to 75% of the RGC, whereas the extension phase covered the remaining portion of the cycle. Temporal parameters are summarized in Table 1. Statistically significant differences between the two locomotion modes were observed for specific parameters. In particular, the duration of the RGC increased in the active mode (*p* < 0.004), along with the duration of the flexion phase (*p* < 0.027), whereas cadence was reduced (*p* < 0.011).

### 3.2. SEMG Patterns

**Figure 2:**
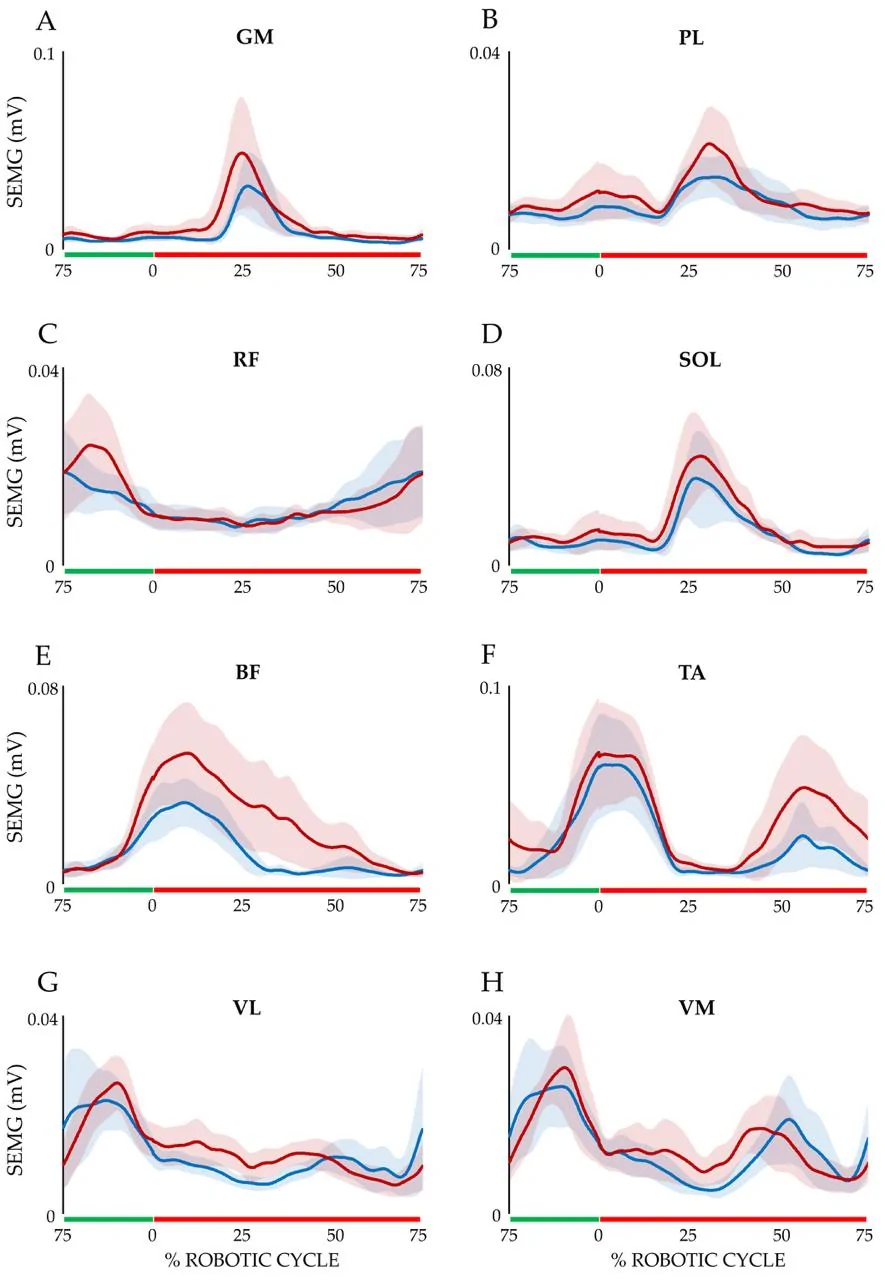
Average SEMG muscle activations among participants in the passive (blue) and active (red) RGC modes. The shaded areas represent 95% confidence intervals. The green and red lines on the x-axis represent the extension and flexion phases, respectively. (**A**) GM (m. gastrocnemius medialis); (**B**) PL (m. peroneus longus); (**C**) RF (m. rectus femoris); (**D**) SOL (m. soleus); (**E**) BF (m. biceps femoris caput longus); (**F**) TA (m. tibialis anterior); (**G**) VL (m. vastus lateralis); (**H**) VM (m. vastus medialis).

Figure 2 shows the average SEMG profiles for each muscle computed among participants, separately for the two RGC modes. Notwithstanding the relatively large intersubject variability in the amplitude of the SEMG signals denoted by the 95% confidence intervals (the colored shaded areas around the solid traces), a general pattern seemingly emerged with respect to the temporal profiles of SEMG activations, which appear rather similar between the two RGC modes.

**Figure 3:**
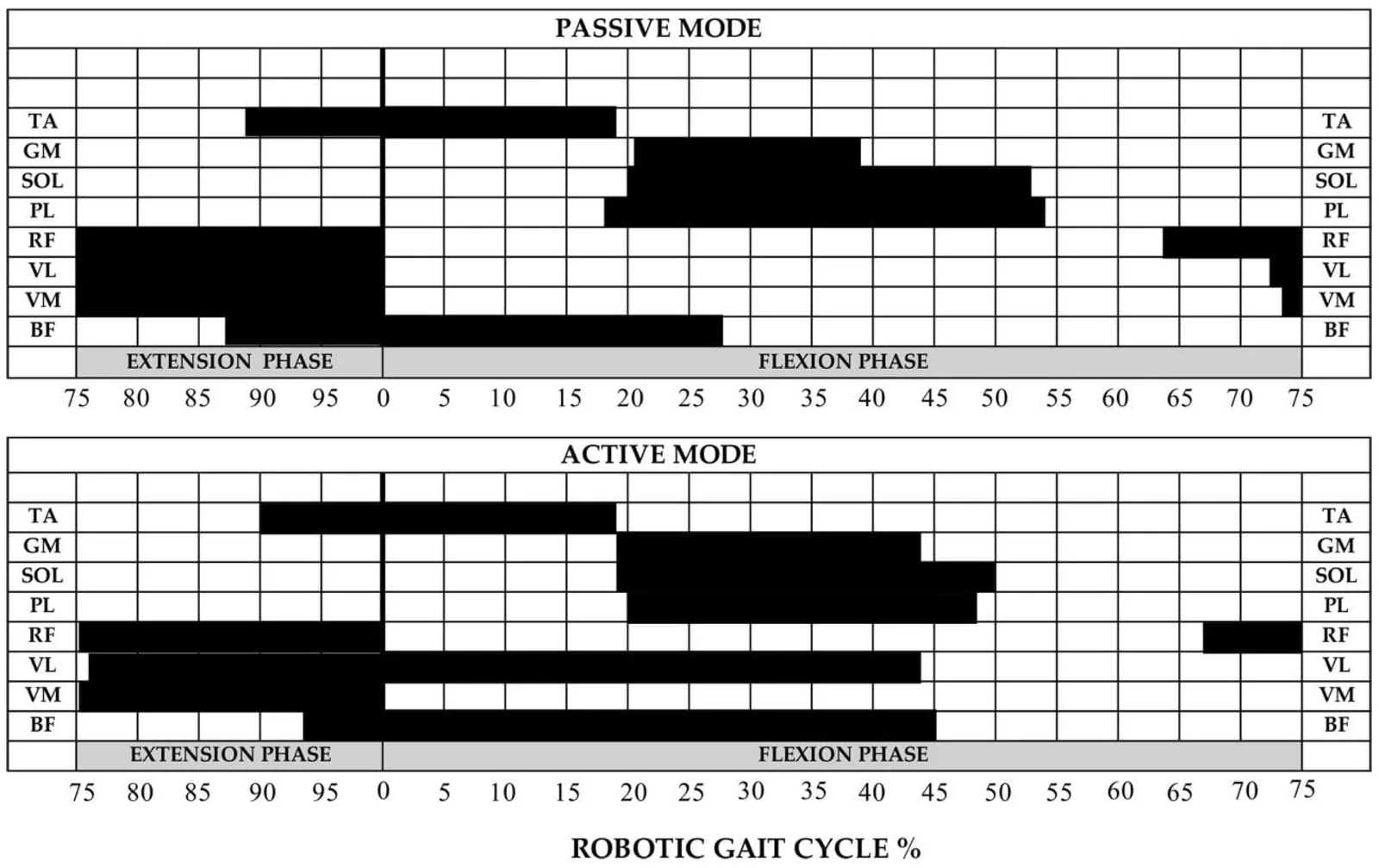
Median SEMG-derived muscle activation timing across participants under passive and active modes, reported as percentage of the RGC. Same muscle abbreviations as in Figure 2.

We investigated this issue further by analyzing, first, qualitatively the timing of muscle activations by defining activation thresholds and then, more quantitatively, with Non-negative Matrix Factorization analysis. Figure 3 illustrates the median values of the muscle activation timing for each muscle in both RGC modes. The median values among subjects were calculated from the mean values of muscle activations observed during each phase of the 10 cycles (11/11). To be included in this figure, each muscle activation had to occur in all subjects in the same segment of each phase and had to last at least 10% of RGC. Median and interquartile ranges of the SEMG activation onsets, offsets, and durations are reported in Supplementary Tables S1 and S2.

As shown, during the passive condition, both TA and BF were active from the final part of the extension phase (TA 88.2%; BF 86.6%) to the initial part of the flexion phase (TA 19.6%; BF 26.6%), whereas distal muscles (GM, SOL, PL) were active during the intermediate part of the flexion phase (GM 21.0–37.6%, SOL 20.0–52.8%, PL 18.2–53.8%). Proximal muscles (RF, VL, VM) were active from the late flexion phase throughout the entire extension phase (RF 64.0–100.0%, VL 72.6–100.0%, VM 74.3–100.0%). A similar pattern was observed during the active mode, but with a longer duration of BF activity from the extension phase (92.8%) to the flexion phase (45.6%). Furthermore, it was observed that in nine out of 11 subjects, the VL was active from the extension phase throughout the flexion phase (from 76.4 to 43.8%), suggesting that the device imposed a greater muscular demand with a coactivation of muscles BF-VL.

### 3.3. Non-Negative Matrix Factorization (NMF)

**Figure 4:**
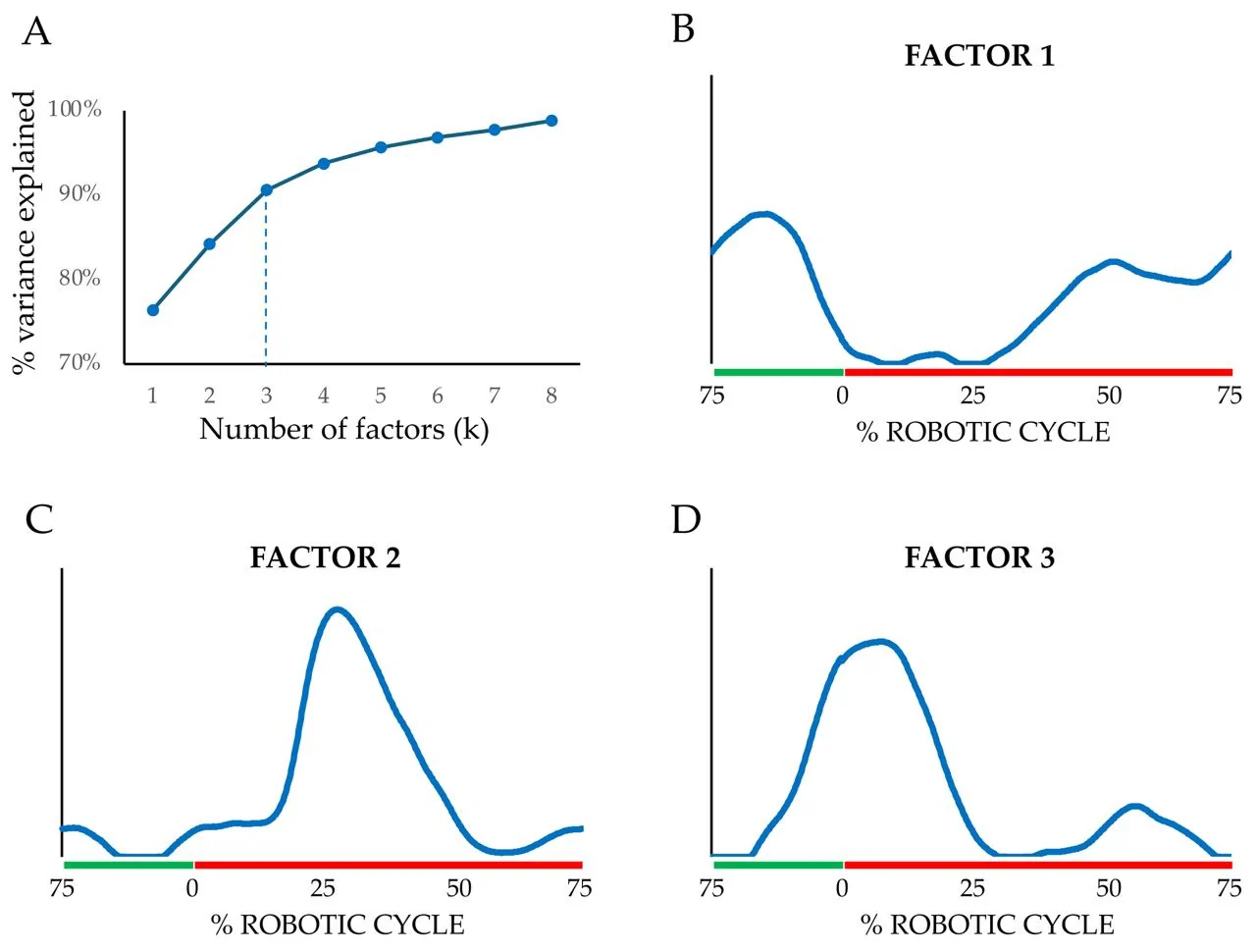
Non-negative Matrix Factorization analysis. (**A**). Percent of variance explained by increasing the number of factors (k) extracted by the Non-negative Matrix Factorization. The vertical dashed line indicates the elbow in the distribution of percent values by which the optimal number of basis waveforms to extract was determined. (**B**–**D**). The three basis waveforms (factors) extracted by the Non-negative Matrix Factorization analysis from the dataset of SEMG muscle activations pooled across muscles, stepping conditions, and participants. The temporal profiles are plotted in relation to the flexion and extension phases of RGC (red and green lines, respectively).

To characterize more quantitatively the temporal profiles of SEMG activity across muscles, RGC conditions, and participants, we performed an NMF analysis on the whole dataset of SEMG waveforms. Using the elbow criterion on a series of NMF models with increasing numbers of factors (k) from one to eight, we found that the optimal number of factors was equal to three, accounting for 90.6% of the variance in the SEMG dataset (Figure 4A).

**Figure 5:**
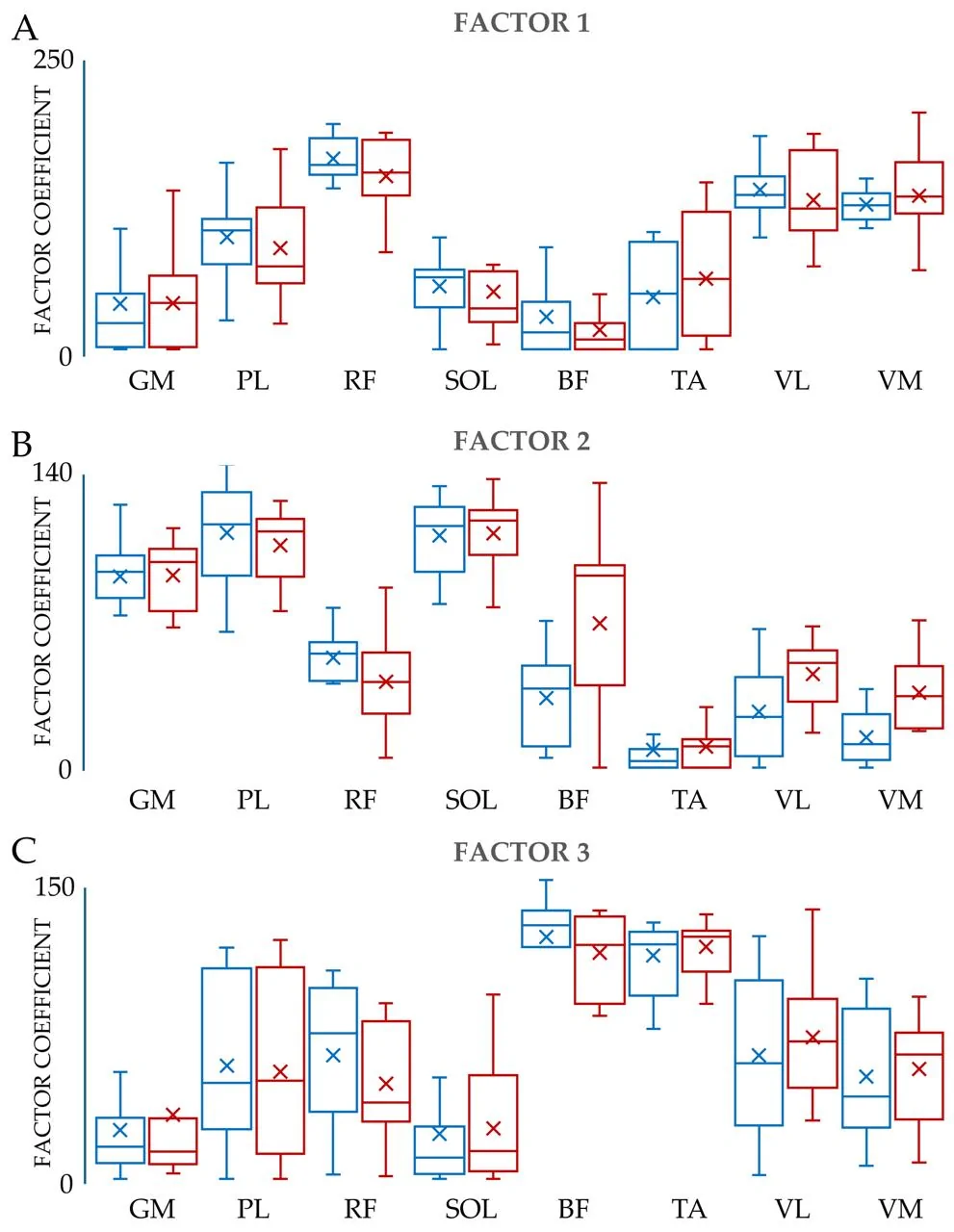
Distributions of the three NMF factor coefficients for the eight muscles recorded. Each box-and-whisker plot represents the distribution of the factor coefficients for each muscle and experimental condition; the horizontal line within each box indicates the median, the box represents the interquartile range, and the whiskers indicate the minimum and maximum values. The blue and red bars refer to the passive and active stepping conditions, respectively. (**A**–**C**) are the distribution coefficients for factor 1, factor 2, and factor 3, respectively. The same muscle abbreviations as in Figure 2.

The three basis temporal waveforms extracted by the NMF analysis are illustrated in Figure 4B–D. The first waveform (panel 4B) represented a temporal activation profile with activity increasing from the middle of the flexion phase and peaking during the first half of the extension phase. This pattern was rather typical of the RF, VM, and VL muscles, as also denoted by the rather high coefficients they showed for this basis waveform (see Figure 5A). The second factor (panel 4C) described a temporal course of activity that peaked at mid-flexion (20–40% of the cycle), which was typically observed in the GM, SOL and PL muscles, which, indeed, showed large coefficient values for this factor (see Figure 5B). Factor 3 (panel 4D) was related to a burst of activity starting at the end of the cycle during the extension phase and extending to the beginning of the flexion phase at 10% of the cycle, mostly observed in the TA and BF muscles, which showed the highest coefficient values for this factor (see Figure 5C). Remarkably, all these patterns of temporal activation were rather similar between passive and active robotic gait conditions (compare the red and blue box-and-whisker plots in Figure 5).

**Figure 6:**
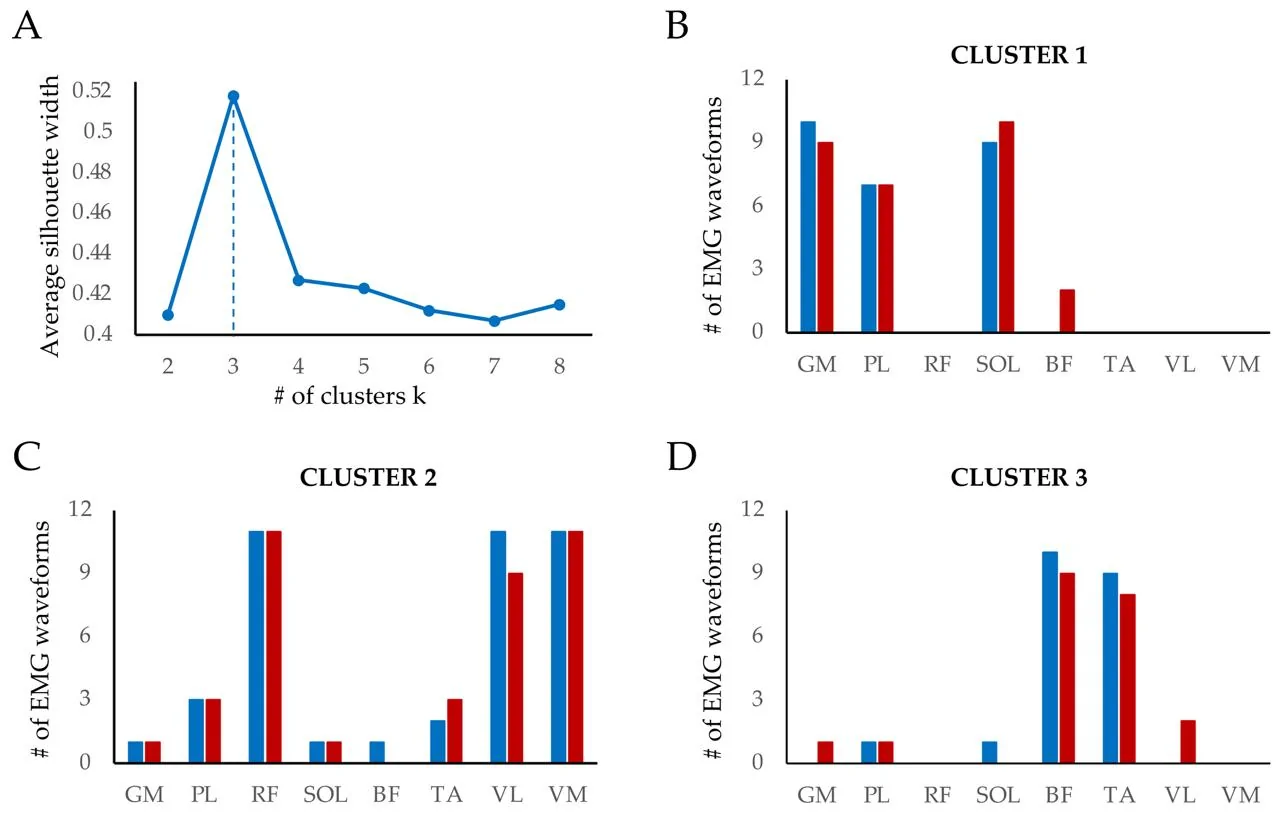
Results of the PAM cluster analysis. (**A**). Identification of the optimal number of clusters by the silhouette method. The vertical dashed line indicates the cut-off for the optimal number, which, according to the silhouette method, corresponded to three clusters. Panels (**B**–**D**) illustrate the distribution of muscle SEMG waveforms among the three clusters. Each bar represents the number of SEMG waveforms of each muscle in the passive (blue bars) and active (red bars) conditions assigned to a given cluster. The same muscle abbreviations as in Figure 2.

Indeed, Wilcoxon signed-rank paired tests comparing coefficients of each muscle between passive and active conditions for each NMF factor did not show statistically significant differences (all *p*-values > 0.05 FDR-corrected), suggesting that the basic temporal profiles of muscle activation were preserved between passive and active conditions. We further investigated the apparent temporal synergies emerging from the analysis of the distributions of the factor coefficients across muscles by performing a cluster analysis with the PAM algorithm on the cosine distance matrix of the factor coefficients. The silhouette method applied to different clustering solutions ranging from 2 to 8 clusters indicated that optimal separation occurred with three clusters (Figure 6A).

The size and medoids of the three identified clusters are reported in Table 2. The first cluster was centered between factor 2 and factor 1, the second between factor 1 and factor 3, and the third was centered mainly on factor 3.

Interestingly, the three clusters more clearly identified the apparent three temporal muscle synergies that emerged from the distributions of the factor coefficients among muscles shown in Figure 5. The first cluster included mainly SEMG waveforms of the GM, PL, and SOL muscles, corresponding to a plantar flexor strategy for foot stabilization and propulsion (Figure 6B). Most of the SEMG waveforms assigned to the second cluster belonged to the RF, VL, and VM muscles, seemingly identifying a temporal synergy of the knee extensors contributing to the extension phase (Figure 6C). The third cluster was populated mainly by SEMG waveforms of the BF and TA muscles, identifying a potential synergy of ankle/knee flexors driving the flexion phase (Figure 6D). Finally, we did not find obvious differences in the distribution of the SEMG waveforms of the eight muscles among the three clusters between the passive and active RGCs (compare the blue and red bars in Figure 6B–D), suggesting that these three muscle synergies were invariant between conditions.

## 4. Discussion

The purpose of the present study was to investigate the neuromuscular responses following locomotor movements generated by an end-effector robot. Specifically, we characterized the temporal patterns of lower-limb muscle activation in a cohort of healthy young adult females during ARL in both passive and active modes. With NMF and cluster analyses, we identified three distinct muscle synergies associated with activations of knee extensor (RF, VL, VM), ankle plantar flexor (GM, SOL, PL), and ankle-knee flexor muscles (TA, BF). We did not observe statistically significant differences in muscle activation timing or muscle synergies between the two ARL modes. Although the analysis of the SEMG amplitudes was beyond the scope of the present study, we must note that some differences in the SEMG activation profiles between the two modes could likely be explained by changes in SEMG amplitude in muscles like the BF, TA, and PL, which contributed more to the active interaction with the robotic device (see Figure 2).

Our experimental protocol included only female participants to avoid potential confounding factors inherent to sex-related biomechanical differences. Specifically, males and females differ in lower-limb (pelvis, hip, and ankle) and trunk kinematics [31], as well as in upper body accelerations during level walking [32]. Consequently, Bruening et al. [31] recommended that sex should be considered when designing and interpreting instrumented gait analysis studies. In addition, these sex-related differences become particularly evident in pathological conditions, such as after total knee arthroplasty [33].

The identification and measurement of RGC depend on the type of robotic device used. Although both exoskeletons and end-effector devices limit the anatomical sites for the application of sensors, exoskeletons allow footswitches to define the phases of the gait cycle [17], whereas end-effector systems can be instrumented with wearable sensors on the malleolus [19], but not on the pelvis or trunk due to the harness constraints. In our experimental protocol, we originally preferred to place a wearable sensor over the footplate to directly capture the kinematics of footplate motion, thereby avoiding the inevitable unsteadiness of the ankle–foot strapped to the footplate. Observational analysis shows that the flexion phase starts from a knee extension position and progresses to knee flexion combined with hip extension. The extension phase follows, with the hip flexed and the knee extended, with the ankle–foot slightly in plantar flexion at the end of RGC. Three-dimensional kinematic and kinetic analyses are required to further characterize the potential subphases of the RGC.

Comparative analyses of lower limb electromyographic patterns have been reported in two studies during overground and ARL [19,22]. Bellitto et al. [19] identified four muscle synergies using PCA, which were associated with balance and positioning of the limbs during the stance-to-swing transition or the stance phase, and reported a reduced neuromuscular contribution with the robotic device. In children with cerebral palsy, Ahn et al. [22] observed lower muscle activations during robot-assisted walking compared to overground walking, with delayed and prolonged activation of the VL, BF, and TA muscles.

The three muscle activation synergies identified by the NMF and the cluster analyses are consistent with the biomechanical constraints imposed by the RGC. Specifically, one synergy is represented by proximal muscles (RF-VL-VM), with VL and VM active during the extension phase, whilst RF was active not only during the extension phase, but also, as a biarticular muscle, during the transition phase. A second synergy corresponds to a distal GM-SOL-PL muscle coactivation pattern. These muscles are not only plantar flexors but also act as stabilizers of the ankle–foot complex. In this regard, it should be noted that the foot is strapped to the footplate, serving as the only distal constraint and, therefore, it requires stabilization. In the third synergy, there is a peculiar contribution of TA and BF both during the initial flexion phase, when the backward motion of the robotic arms causes knee flexion with ankle dorsiflexion, and the last extension phase, when both BF and TA are stretched, suggesting an eccentric contraction.

According to Maranesi et al. [34], a standard RAGT protocol using an end-effector robot consists of five sessions performed in passive mode, followed by 15 sessions in active-assistive mode. The latter mode is particularly important because it promotes active participation of the patients in the training program. Therefore, we recorded lower-limb muscle activity during both conditions to investigate whether a moderate level of assisted effort (50% of body weight) induced changes in the temporal patterns of SEMG activity that differed significantly from those observed in passive mode. Previous studies have shown that the amplitude of SEMG signals from lower-limb muscles increases with walking speed at specific phases or subphases of overground gait [35], whereas the relative timing of muscle activation remains consistent across different walking speeds [35,36] and when carrying additional weight [37]. In our study, the target velocity was kept constant across both modes. However, in active mode, the duration and cadence of the RGC showed significant changes, but muscle activation timing remained unchanged compared with passive mode. The only observed difference was the co-activation of the biceps femoris (BF) and vastus lateralis (VL), which may reflect a strategy to stabilize the knee during the first half of the flexion phase.

In our study, RGC was not directly compared with overground walking due to differences in limb trajectories, center-of-mass control, and overall posture. Because of these differences, in fact, walking in exoskeletons or with the feet strapped to footplates cannot be considered physiological gaits and therefore cannot be compared directly. Nonetheless, robotic training is valuable for stimulating spinal circuits and CPGs as it facilitates interneuron activation located between CPGs and motoneurons [38]. The goal of robotic rehabilitation should be to train spinal circuits in a different modality rather than to replicate the overground gait. In our study, the identification of a limited number of muscle synergies underlying locomotion compared to those reported for overground walking [4] suggests that the central nervous system during robotic locomotion may simplify further motor control by activating specific groups of muscles as coordinated rhythmic locomotor patterns. A similar methodological approach can be observed in physical therapy interventions aimed at restoring motor system function in patients with spinal cord injury. These interventions are part of Activity-Based Therapies (ABT) [39], which are designed to promote the activation and reorganization of spinal locomotor networks through repetitive and task-specific movements. Similarly, athletic training often involves high-intensity, repetitive, and rhythmic movements, such as sprinting, plyometric exercises, and cycling, which are used to optimize the efficiency of spinal neural networks involved in movement control [40]. These types of training can improve the automaticity of locomotor patterns by strengthening spinal circuits that can operate with a reduced reliance on continuous supraspinal input [41,42].

## 5. Limitations

The number of subjects participating in this study was limited, but the subjects are quite homogeneous, have similar anthropometric characteristics, are the same sex, and are not involved in competitive sports activities. Moreover, the locomotor task was simple, repetitive, and did not require a particular effort or ability.

Another limitation is that we were unable to quantify the level of participation during the two locomotor tasks. However, we have provided evidence that there are statistical differences between some key parameters of RGC (duration, cadence, and flexion phase), indicating a different involvement during the execution of the two modalities of robotic walking.

## 6. Conclusions

Jacquelin Perry [43], along with previous studies [44,45], emphasized the importance of timing in clinical gait analysis, providing normative descriptions for overground walking. In interpreting pathological gait, the relative timing of muscle activity compared to normal function is highly significant, allowing the identification of abnormal agonist–antagonist synchronizations, prolonged or shortened EMG burst durations, overactivations, and co-activation phenomena. Our study gives some original insight into the functioning of the neuromuscular system during robotic gait and highlights the interest of comparing physiological and pathological muscle patterns. Furthermore, the study provides preliminary data on the timing of muscle activation during robot-assisted locomotion with an end-effector gait trainer in a cohort of healthy young adult females. Since previous studies have shown that the timing of SEMG activation patterns throughout the gait cycle is similar between adult males and females [46], our preliminary findings may potentially be extended to a male population, considering, however, the evident biomechanical differences related to sex in gait. In any case, a study that applies the same methodology to a group of male participants is currently underway to further investigate this aspect.

This study supports the integration of clinical gait analysis, particularly surface electromyography, into robot-assisted gait training protocols through the evaluation of patients before and after the intervention. The main objective should be to promote the recovery of muscle patterns such as those of healthy individuals walking on a robotic device. True improvement will be obtained when the SEMG patterns of our patients approach the normative data.

## Figures and Tables

**Table 1:** Median and interquartile ranges of the temporal parameters of the robotic gait cycle (RGC) among participants. *p* values and Rosenthal’s effect sizes (*r*) of Wilcoxon tests for paired samples are reported for the statistically significant differences between active and passive modes (*p* < 0.05).

	Flexion Phase	Extension Phase	RGC	Flexion Phase	Extension Phase	Cadence
	%	%	SEC	SEC	SEC	steps/min
**Passive**	74.80 (74.55–74.90)	25.30 (25.20–25.50)	2.38 (2.37–2.39)	1.78 (1.77–1.79)	0.60 (0.59–0.60)	25.20 (25.15–25.35)
**Active**	74.90 (73.85–75.30)	25.10 (24.80–25.30)	2.58 (2.54–2.66)	1.95 (1.91–1.99)	0.64 (0.61–0.69)	23.30 (22.60–23.75)
***p* value**	n.s.	n.s.	0.034	0.043	n.s.	0.036
**Rosenthal’s *r***	-	-	0.559	0.455	-	0.503

**Table 2:** Cluster sizes and medoids for the three clusters identified by the PAM analysis in the space defined by the three factors extracted by the NMF analysis.

Cluster #	Size	FACTOR 1	FACTOR 2	FACTOR 3
1	54	46.71	130.68	26.00
2	80	179.73	53.54	80.53
3	42	38.94	39.83	125.43

## Data Availability

The data that support the findings of this study are available from the corresponding author upon reasonable request.

## Supplementary Materials

The following supporting information can be downloaded at: https://www.mdpi.com/article/10.3390/s26216960/s1: Table S1: Distributions among participants of onset, offset and duration of muscle activations during passive mode of assisted robotic gait. Values are reported as percent of the robotic gait cycle. GM (m. gastrocnemius medialis), PL (m. peroneus longus), RF (m. rectus femoris), SOL (m. soleus), BF (m. biceps femoris caput longus), TA (m. tibialis anterior), VL (m. vastus lateralis) and VM (m. vastus medialis). Table S2: Distributions among participants of onset, offset and duration of muscle activations during active mode of assisted robotic gait. Values are reported as percent of the robotic gait cycle. Same muscle abbreviations as Table S1.
