# Supplementary material for: Muscle Activation Timing and Synergies During End-Effector Robot-Assisted Gait: A Surface EMG Study
Source: Sensors (Basel). 2026 Nov 4;26(21):6960. doi: 10.3390/s26216960 (PMC13766348; doi:10.3390/s26216960)
Supplement: Supplementary file 1 [file sensors-26-06960-s001.zip › sensors-4545021-supplementary.pdf]

Table S1. Distributions among participants of onset, offset and duration of muscle activations during passive mode of assisted robotic gait. Values are reported as percent of the robotic gait cycle. GM (gastrocnemius medialis), PL (m. peroneus longus), RF (m. rectus femoris), SOL (m. soleus), BF (m. biceps femoris caput longus), TA (m. tibialis anterior), VL (m. vastus lateralis) and VM (m. vastus medialis).

MUSCLE	% ONSET		% OFFSET		% DURATION	
	MEDIAN	IQR (25-75)	MEDIAN	IQR (25-75)	MEDIAN	IQR (25-75)
TA	88.2	(82.1-94.2)	19.6	(16.4-20.4)	29.8	(24.5-33.5)
GM	21.0	(19.3-22.4)	37.6	(36.0-50.0)	17.0	(14.7-27.5)
SOL	20.0	(18.6-21.0)	52.8	(41.7-54.6)	33.0	(21.5-35.9)
PL	18.2	(16.6-19.0)	53.8	(50.5-58.0)	36.2	(33.6-40.4)
RF	64.0	(44.5-68.5)	100.0	(94.3-100.0)	36.0	(29.4-47.5)
VL	72.6	(64.0-75.4)	100.0	(95.0-100.0)	27.2	(23.5-31.7)
VM	74.3	(71.3-75.3)	100.0	(100.0-100.0)	24.9	(20.6-25.9)
BF	86.6	(80.0-90.3)	26.6	(24.6-31.7)	45.0	(38.8-50.7)

Table S2. Distributions among participants of onset, offset and duration of muscle activations during active mode of assisted robotic gait. Values are reported as percent of the robotic gait cycle. Same muscle abbreviations as Table S1.

MUSCLE	% ONSET		% OFFSET		% DURATION	
	MEDIAN	IQR (25-75)	MEDIAN	IQR (25-75)	MEDIAN	IQR (25-75)
TA	90.2	(67.2- 92.2)	18.8	(18.6-20.0)	29.6	(26.3-50.3)
GM	18.8	(15.2-20)	43.8	(37.4-49.1)	29.2	(19.6-31.8)
SOL	18.6	(17.1-19.4)	49.6	(44.0-51.1)	32.4	(26.5-34.6)
PL	20.4	(19.3-20.7)	47.1	(40.2-51.9)	24.2	(16.2-31.1)
RF	67.2	(59.3-75.4)	98.2	(95.7-100.0)	28.4	(22.7-35.9)
VL	76.4	(73.3-79.7)	43.8	(25.4-48.4)	68.1	(54.2-68.6)
VM	75.2	(72.9-78.4)	100	(100.0-100.0)	22.6	(21.3-26.1)
BF	92.8	(89.7-94.0)	45.6	(33.0-52.7.0)	51.2	(43.9-61.5)
